# Pharmacologically-induced Recreational Priapism: Case Report and Review

**DOI:** 10.5811/cpcem.2020.8.47763

**Published:** 2020-10-09

**Authors:** Grace Kunas, Janet A. Smereck, Diana Ladkany, Jonathan E. Davis

**Affiliations:** * Abrazo Health, Department of Emergency Medicine, Goodyear, Arizona; † Georgetown University Medical Center, Department of Emergency Medicine, Washington, District of Columbia

**Keywords:** ischemic priapism, phosphodiesterase inhibitor, intracorporal injection

## Abstract

**Introduction:**

Priapism, a time-sensitive urologic emergency, is associated with hematologic disorders, malignancies, trauma, pharmaceuticals, and recreational drugs.

**Case Report:**

A 51-year-old male presented with 36 hours of priapism after recreational use of nonprescribed pharmaceuticals including an oral phosphodiesterase inhibitor and intracorporally injected erectile medications, together with unspecified quantities of cocaine and alcohol. Venous blood gas confirmed ischemic priapism. Detumescence was achieved with intracavernosal phenylephrine injection, aspiration, and irrigation.

**Conclusion:**

This case highlights the risk that recreational use of vasoactive medications by patients who seek to prolong sexual activity may lead to delayed presentation for ischemic priapism.

## INTRODUCTION

Numerous factors may lead to priapism, defined as penile erection in the absence of active sexual stimulation that persists for a minimum of four hours due to persistent engorgement of the corpora cavernosa and disruption of normal detumescence mechanisms.^1^ Priapism is a time-sensitive urologic emergency that is relatively rare in the general population; priapism disproportionately affects patients with hematologic and neurologic disorders, notably sickle cell disease and spinal cord injury, and is associated with a number of pharmaceuticals and recreational drugs.^2^,^3^ Although the mean duration is just over two hours per episode, delayed presentation can be associated with use of medications intended to stimulate potency or manage erectile dysfunction.^1^ There are two main subtypes of priapism: ischemic (low inflow), and non-ischemic (high inflow). Ischemic priapism, the most frequent subtype, requires emergent intervention to avoid future complications including tissue necrosis, fibrosis and scarring, and subsequent erectile dysfunction.^1^,^2^

## CASE REPORT

A 51-year-old male presented to the emergency department (ED) with a chief complaint of persistent erection that had been present for approximately 36 hours after he ingested 20 milligrams (mg) of tadalafil followed by penile self-injection with approximately one milliliter (mL of Trimix, a compound containing alprostadil, papaverine, and phentolamine. He had no history of erectile dysfunction. Neither medication had been prescribed to him, and his intention was to achieve a sustained penile erection to facilitate prolonged sexual activity with a group of people. There was concomitant use of an unspecified quantity of alcohol as well as intranasal cocaine. The patient reported mild discomfort and some difficulty passing urine due to his prolonged erection, but reported he was still able to void. He denied hematuria, dysuria, or penile discharge. There was no past history of malignancy or hematologic disorders. His medical issues included stable human immunodeficiency virus (HIV) disease and reported compliance with antiretroviral medication (emtricitabine-tenofovir); there was no history of hypertension.

On examination the patient did not appear to be in distress; his demeanor was consistent with mild intoxication, but he was fully alert and oriented to person, place, time, and situation. His initial vital signs were notable for hypertension, with a blood pressure of 161/104 millimeters of mercury (mm Hg), heart rate of 88 beats per minute, and an afebrile temperature of 36.1 degrees Celsius. His penis was erect and engorged; glans and spongiosum were soft without tenderness to palpation. The examiner was unable to bend the shaft due to rigidity. The testes and vas were nontender.

Initial laboratory evaluation included normal hemogram and renal function; urinalysis showed three white blood cells and six red blood cells per high-powered field. Urologic consultation was requested and ice packs were applied to the penile shaft for several minutes without response. Aspiration of the corpora was performed. Penile venous blood gas (VBG) was remarkable for pH of 7.00 (reference [ref] range 7.31–7.41), partial pressure of carbon dioxide of 88.4 mm Hg (ref range 40–52 mm Hg), calculated venous oxygen saturation of 8.0 % (ref range 75%), and lactic acid level of 14.4 milllimoles per liter (mmol/L) (ref range 0.5–2.2 mmol/L), consistent with ischemic priapism. After local anesthetic, corporal injection of phenylephrine 500 μg/mL, diluted in 10 mL of normal saline, followed by aspiration and irrigation with an additional 10 cubic centimeters (cc) of sterile saline, resulted in gradual detumescence. A compression dressing was then applied to the penile shaft. The patient reported no discomfort after the procedure; blood pressure on discharge was normalized to 124/81 mm Hg. The patient was given instructions to remove the pressure dressing in 24 hours and abstain from sexual intercourse for a minimum of seven days.

On follow-up in urology clinic 11 days after his presentation to the ED, the patient reported ability to achieve normal erections; he was noted to have minimal ecchymosis at the base of the penile shaft. He was advised to refrain from using erectogenic medications in the future.

## DISCUSSION

Penile erections are a result of vasodilation in the corpora cavernosa, mediated by inhibition of the cyclic guanosine monophosphate (cGMP) hydrolyzing enzyme phosphodiesterase-5 (PDE5). With ischemic priapism, venous outflow is reduced and venous congestion leads to diminished or loss of arterial inflow.^1^ Tadalafil is one of several available PDE5 inhibitors. Trimix, a compound intended for intracorporal injection (ICI), consists of alprostadil, phentolamine, and papaverine. The three pharmaceuticals act in different ways to stimulate penile vasodilation. Alprostadil acts directly to stimulate the production of cyclic adenosine monophosphate (cAMP), which like cGMP leads to penile tumescence from smooth muscle relaxation. Phentolamine is an antagonist of alpha adrenergic receptors, resulting in further vascular dilation, while papaverine inhibits several subclasses of PDEs, causing cGMP and cAMP accumulation.^4^

CPC-EM Capsule

What do we already know about this clinical entity?

*Ischemic priapism may result from pharmaceuticals including phosphodiesterase inhibitors, injected vasodilators, psychotropics, and alpha-receptor antagonists*.

What makes this presentation of disease reportable?

*A 36-hour episode of ischemic priapism after combined use of oral and cavernosally injected erectile agents was treated emergently without long-term complications*.

What is the major learning point?

*Use of erectile medications can result in delayed presentation for ischemic priapism, best managed with sympathomimetic agents injected into the corpora cavernosa*.

How might this improve emergency medicine practice?

*This report adds to the literature on priapism causes, management and outcomes, highlighting the adverse consequences of recreational use of erectile medications*.

While the above described medications are intended to facilitate erections for a safe duration in persons with erectile dysfunction, this case describes a patient with normal erectile function who recreationally misused a combination of pharmaceuticals resulting in a prolonged, unsafe erection. A retrospective study of 169 priapism encounters highlighted growing recreational use of ICIs within a metropolitan community, finding nearly 50% of cases to be due to ICIs.^5^ Numerous cases of priapism with various medication classes, in addition to ICIs and PDE inhibitors, have been documented. Psychotropic medications implicated in recreational misuse include the following: serotonin specific reuptake inhibitors, most notably trazodone; amphetamines; pregabalin; and typical and atypical antipsychotics (due to alpha-blocking characteristics).^6^–^9^ Tamsulosin, due to alpha blocking effects, as well as alpha-receptor antagonist antihypertensives have been associated with ischemic priapism.^10^ Priapism associated with cocaine use has long been observed.^11^ Some herbal supplements marketed for sexual enhancement have been reported in cases of prolonged priapism. *Tribulus terrestris* has been associated with protracted episodes of priapism (between 48–72 hours), as well as the herbal product yohimbine, ranging between 20–72 hours in case reports.^12^,^13^ Pharmaceutical agents reported in association with priapism are summarized in the following table.

Regardless of etiology, ischemic or low-flow priapism is a urologic emergency requiring prompt intervention. Diagnosis should be accomplished through history and physical exam, followed by corporal VBG. Venous blood in ischemic priapism is characterized by an acidotic pH (below 7.1).^2^ Hematologic studies can be obtained and duplex ultrasonography can be done to assess arterial blood flow but is not required.^2^ Once ischemic priapism is suspected, management should begin promptly to prevent compartment syndrome and future fibrosis, which then may contribute to permanent impotence. Urologic input is appropriate if feasible. Initial conservative measures may include application of ice packs to the genital area and asking the patient to perform vigorous physical exercise of the lower limbs.^14^ Aspiration of blood from the corpora cavernosa is used both for blood gas analysis and to remove blood to relieve cavernosal compartment pressure, which then should be followed by saline irrigation.

If detumescence does not occur, the next step is ICI of an alpha-adrenergic sympathomimetic; this is generally preceded by local anesthetic or nerve block.^2^ The preferred sympathomimetic agent is phenylephrine hydrochloride 10 mg/mL; 0.1–0.5 mL (1–5 mg) is diluted in 10 cc normal saline to give a concentration of 100–500 μg/mL, injected at a rate of 1 mL over three to five minutes until detumescence is achieved. Diluted epinephrine 1:10,000 solution may be used if phenylephrine is unavailable.^2^,^15^ Sonographic guidance may be used to facilitate cavernosal needle placement but is not a requirement. Oral pseudoephedrine as well as subcutaneous terbutaline are other options, but are less effective and not recommended for priapism of duration greater than four hours. If these measures fail, a surgical procedure to implant shunts may need to be performed.^2^

By contrast, non-ischemic priapism, which is rarer and usually a result of trauma or congenital arterial malformation, occurs from unregulated arterial inflow to the corpora cavernosa, creating an arteriolar-sinusoidal fistula. Due to constant high inflow of arterial blood, the corpora cavernosa is not at risk for ischemia and the condition is, therefore, non-ischemic priapism. Non-ischemic priapism is generally painless; treatment is routinely conservative, as most cases resolve spontaneously.^2^

## CONCLUSION

Numerous pharmaceutical agents have been associated with priapism; in addition to medications used to treat erectile dysfunction, several psychotropic medications have been implicated. Recreational use of vasoactive pharmaceuticals with the intention of prolonging sexual activity can lead to delayed presentation for ischemic priapism. In particular, patients who misuse intracorporally injected medications present four hours later on average than those with priapism from other etiologies, more frequently have comorbid HIV disease, and more commonly co-ingest recreational drugs and alcohol, which can exacerbate priapism and likely contribute to delayed presentation.^3^ A patient presented with an episode of ischemic priapism of approximately 36 hours in duration after use of diverted erectogenic pharmaceuticals, likely exacerbated by concomitant use of cocaine. The patient underwent active intervention in the ED to achieve detumescence, with no apparent permanent sequelae. This case adds to the growing literature on pharmacologically induced priapism. Emergency clinicians should be aware of the potential for misuse of medications prescribed for erectile dysfunction and the need for active management to achieve a favorable outcome.

## Figures and Tables

**Table:** Pharmaceutical agents associated with priapism.

Category	Agent(s)
Erectile dysfunction medications	ICI agents, PDE inhibitors
Antidepressants/ SSRIs	Paroxetine, citalopram, trazodone
Antipsychotics	Atypical and typical (haloperidol, chlorpromazine, quetiapine, clozapine, olanzapine, risperidone)
Amphetamines	Methylphenidate, dexmethylphenidate, lis-dexamphetamine
Anti-epileptic/pain management	Pregabalin
Alpha-adrenergic blockers	Antihypertensives (terazosin), tamsulosin
Herbal products	*Tribulus terrestris*, yohimbine
Recreational drugs	Cocaine, ethanol

*ICI*, intracorporal injection; *PDE*, phosphodiesterase; *SSRI*, selective serotonin reuptake inhibitor.

## References

[1] Cuartas J, Sandoval-Salinas C, Martínez J (2019). Treatment of priapism secondary to drugs for erectile dysfunction. Adv Urol.

[2] Vilke GM, Harrigan RA, Ufberg JW (2004). Emergency evaluation and treatment of priapism. J Emerg Med.

[3] Scherzer ND, Reddy AG, Le TV (2019). Unintended consequences: a review of pharmacologically induced priapism. Sex Med Rev.

[4] Nehra A (2007). Oral and non-oral combination therapy for erectile dysfunction. Rev Urol.

[5] Zhao H, Berdahl C, Bresee C (2019). Priapism from recreational intracavernosal injections in a high-risk metropolitan community. J Sex Med.

[6] Saghafi O, Kao A, Druck J (2014). Recurrent priapism from therapeutic quetiapine. West J Emerg Med.

[7] Bhat IA, Shannon KD, Ara A (2015). Ninety-six hours ordeal of priapism induced by paroxetine: a case report and literature review. Int J Psychiatry Med.

[8] Compton MT, Miller AH (2001). Priapism associated with conventional and atypical antipsychotic medications: a review. J Clin Psychiatry.

[9] Karancı Y (2020). Priapism associated with pregabalin. Am J Emerg Med.

[10] Marconi M, Pavez P, San Francisco I (2019). Priapism induced by use of tamsulosin: a case report and review of the literature. Arch Ital Urol Androl.

[11] Altman AL, Seftel AD, Brown SL (1999). Cocaine associated priapism. J Urol.

[12] Campanelli M, De Thomasis R, Tenaglia RL (2016). Priapism caused by ‘Tribulus terrestris.’. Int J Impot Res.

[13] Myers A, Barrueto F (2009). Refractory priapism associated with ingestion of yohimbe extract. J Med Toxicol.

[14] Gravel J, LeBlanc C, Varner C (2019). Management of priapism with a trial of exercise in the emergency department. CJEM.

[15] Roberts J, Price C, Mazzeo T (2009). Intracavernous epinephrine: a minimally invasive treatment for priapism in the emergency department. J Emerg Med.

